# OP81 Outcome-Based Managed Entry Agreements For Rare Disease Therapies: Learnings From The Belgian Experience

**DOI:** 10.1017/S0266462325101359

**Published:** 2025-12-29

**Authors:** Alessandra Blonda, Karen Facey, Inneke Van de Vijver, Yvonne Denier, Isabelle Huys, Steven Simoens

## Abstract

**Introduction:**

The rising costs of rare disease therapies pose significant challenges for health technology assessment (HTA) decision-makers and payers, particularly given the limited clinical evidence available at the time of appraisal. This has created a growing need for outcome-based managed entry agreements (OBMEAs) that link post-launch evidence generation to appropriate payment models. This study examined the implementation of OBMEAs in Belgium and offers best practices to optimize their use globally.

**Methods:**

A document analysis was conducted of managed entry agreements (MEAs) for all rare disease therapies reimbursed between January 2012 and August 2024. Through a standardized template, OBMEAs were identified and classified based on type of uncertainties included and outcomes to be collected. HTA reports were analyzed to assess the impact of clinical and real-world evidence (RWE) on the reassessment of these therapies at the end of the OBMEA.

**Results:**

The Belgian payer implemented outcome-based managed entry agreements (OBMEAs) for 57 orphan drugs, including five advanced therapy medicinal products. All OBMEAs followed a coverage with evidence development scheme, primarily addressing budget impact (32%) and efficacy (27%). The median duration was 24 months, with some agreements extended or renewed up to five times. Only eight percent of OBMEAs resulted in a definitive listing. RWE was frequently rejected during reassessment due to concerns about validity and incomplete registries. Key recommendations include establishing clearer data collection protocols and improving transparency at multiple levels to enhance the effectiveness of these agreements.

**Conclusions:**

The findings highlight the challenges of implementing outcome-based MEAs in a manner that truly addresses long-term uncertainties. Adopting the best practices outlined in this study could support OBMEAs to fulfill their promise of balancing sustainability with timely patient access to innovative therapies.

